# Blessings or burdens: an Interpretative Phenomenological Analysis (IPA) study on the motivations and their impact on end-of-life caregiving among Asian family caregivers

**DOI:** 10.1186/s12904-020-00638-6

**Published:** 2020-08-20

**Authors:** Geraldine Tan-Ho, Ping Ying Choo, Paul Victor Patinadan, Casuarine Xinyi Low, Andy Hau Yan Ho

**Affiliations:** 1 Psychology Programme, School of Social Sciences, Nanyang Technological University, Singapore, Singapore; 2 Centre for Population Health Sciences, Lee Kong Chian School of Medicine, Nanyang Technological University, Singapore, Singapore; 3 Palliative Care Centre for Excellence in Research and Education, Singapore, Singapore

**Keywords:** Palliative care, Caregiver, Motivations, Wellbeing, Meaning, Burnout, Resilience, Self-determination, End-of-life, Qualitative research

## Abstract

**Background:**

While the impact of family caregiving has been well-documented, many of such studies center on investigating external factors such as socioeconomic status, accessibility to resources and availability of social support as the primary causation of caregiver wellbeing outcomes. This paper explores the motivations that drive family caregivers in supporting their family members at the end-of-life, and critically examines how internal appraisal processes of such motivations can both positively and negatively impact their wellbeing.

**Methods:**

This study adopted an interpretative phenomenological analysis (IPA) to investigate the motivations and internal appraisal processes of Asian family caregivers in Singapore who were tending to a dying family member. Qualitative dyadic interview data (*N* = 20) was drawn from a larger Randomized Controlled Trial for a novel Family Dignity Intervention (FDI) for palliative care patients and their families. The sampling population consisted of participants aged 21 and above who were identified to be the primary caregivers of older palliative care patients with a prognosis of less than 12 months. Data collection was conducted in the homes of patients and family caregivers.

**Results:**

Findings revealed six themes that could either nurture or diminish caregiver wellbeing: 1) Honoring Fidelity (caregivers were motivated to commit to their caregiving roles in order to avoid regret), 2) Alleviating Suffering (caregivers were motivated to relieve their family member’s pain), 3) Enduring Attachment (caregivers were motivated to spend time together with their family member), 4) Preserving Gratitude (caregivers were motivated to express their appreciation to their family member by caregiving), 5) Navigating Change (caregivers were motivated to adapt accordingly to changes in the illness trajectory) and 6) Reconciling with Mortality (caregivers were motivated to respond accordingly to their family member’s prognosis). The final theme of the Wellbeing Determinant is posited as an indication of self-determination, and is conjectured to influence how caregiving motivations are appraised by the caregiver.

**Conclusion:**

Fulfilling and enhancing one’s sense of self-determination appears central to infusing one’s caregiving motivations with positive meaning, and consequently nurturing one’s wellbeing in the end-of-life caregiving journey. These findings are discussed with recommendations for healthcare professionals working with family caregivers of palliative care patients.

## Background

The experience of an end-of-life (EoL) family caregiver can be likened to a paradox – what could evoke a sense of pleasure, appreciation and gratitude could also bring about feelings of anxiety, distress and pain. While some have related the caregiving journey to the metaphor of ascending a mountain [1], the expedition of an EoL family caregiver usually spans beyond merely a couple of days, weeks or months; they must navigate the peaks of diagnosis to prognosis and eventually death and bereavement, in what often unfolds into a lifelong climb.

The role of the EoL family caregiver is often multifaceted and interminable. Daily duties involve managing medical regimes, traversing the healthcare system, and taking charge of other dependents, alongside providing physical, mental and emotional support throughout the illness trajectory [2, 3]. Many family caregivers are rarely equipped with formal or adequate training, and nor do they possess sufficient resources and skills, before they find themselves embroiled in EoL caregiving responsibilities. Family caregivers must also process and manage a multitude of thoughts and emotions as they come to terms with the changes, and sometimes losses, in their personal lives [4].

This complex experience is not limited to a minority of people. Despite strong global advancements in medical technology and healthcare systems, older populations remain highly susceptible to chronic and terminal morbidities that are incapacitating [5]. In the United States alone, over 40 million caregivers tend to their ailing family members annually [6], while an estimated 80% of patients in Europe requiring long-term care are attended to by informal caregivers [7]. With the anticipated number of older adults in the world soaring to 2 billion by the year 2050 [8], there will certainly be a surging demand for family caregivers to relieve the ensuing resource strains on healthcare settings.

The impact of family caregiving stressors has been well-documented [9–12], with various studies exploring caregiver burnout and its effects on the community and society. Complementing these studies are literature that reveal characteristics of resilience and transformational growth displayed by family caregivers in adversity [13–16]. The common thread that impacts both caregiver burnout and resilience appears to be a lack of, or adequate, *coping*; a process that requires the individual to constantly change efforts in both thoughts and behaviors in order to manage internal or external demands that are considered stressful [17]. Despite this indication that one’s psychological resources are key to maintaining one’s wellbeing, many studies often consider external factors such as socioeconomic status, accessibility to resources and availability of social support as the primary causation for the degree of caregiver wellbeing. Thus, these studies often recommend pragmatic interventions that focus on improving external circumstances accordingly.

### Perception, emotion and motivation of the family caregiver

While it is undoubtedly beneficial to mitigate tangible stressors, one must not lose sight of the magnitude of a person’s internal perception and appraisal of the demands of caregiving. This perception and appraisal embody one’s subjective caregiver burden – observed in reviews of over 50 caregiver studies to pose deep-seated implications on caregivers’ quality of life, levels of depression and anxiety, and stress coping [18, 19]. Campbell et al. [20] later confirmed this observation in a study that evaluated multiple variations in the caregiver experience; subjective caregiver burden was repeatedly identified as the primary indicator for caregiver stress.

It was around the same time that Folkman and Moskowitz [17] discovered that caregivers experience positive emotions alongside negative emotions during stressful events. They put forth the tenet of meaning-focused coping, in which caregivers derive mental and emotional sustenance (thus effecting positive emotions) in challenging circumstances by deferring to their beliefs, values and existential goals. Folkman and Moskowitz found that meaning-focused coping is an intrapsychic process of 1) discovering benefits in caregiving, 2) reminding oneself of such benefits, 3) setting goals that inspire a sense of mastery and competence, 4) realigning priorities in view of changes and 5) infusing ordinary events with positive meaning. Folkman [21] further attested that meaning-focused coping exists alongside negative appraisals in a caregiver’s stress process in order to reinstate physiological and psychological resources during stressful events.

Given the importance of psychological appraisal, it is reasonable to postulate that gaining insight into a caregiver’s beliefs, values and goals that stimulate them in caregiving (defined as *motivations* in this paper) would yield valuable information that could be used to enhance meaning-focused coping. Such interventions would target the essence of a person’s self-concept – that is, the individual’s perception of who they are and what they believe in – and transcend current cursory social, mental and emotional symptom management for caregiver stress.

### Bridging the research gap in Asian caregiving

While the multidimensional nature of caregiving burden and coping is not confined to any specific culture, there are distinctive elements that bear influence on the Asian caregiving experience. Family takes precedence in Asian societies, with strongly inculcated values and expectations of filial piety and filial responsibility placed upon family members [22, 23]. A study conducted in Singapore, a multi-ethnic society that is predominantly Chinese, found that family caregivers who internalized and prioritized societal expectations as motivations over their personal wellbeing most often faced internal conflict and were highly likely to experience difficulties in maintaining their mental health, familial ties and caregiving duties [24]. This is corroborated by the evolving attitudes towards such Confucian rules – younger Asian generations no longer perceive absolute submission or complete obedience to the family as instrumental values in a modern and globalized society [25], and it would be valuable to further understand how these complexities might manifest in a family caregiver’s motivations.

This paper aims to contribute to and grow the current body of knowledge for Asian EoL family caregivers by answering the following research questions: 1) what are the internalized motivations (*defined here as unconsciously assimilated beliefs and values into one’s attitudes or behaviors*) of Asian family caregivers? 2) How might these motivations affect the way they respond to caregiving, and impact their wellbeing? 3) How can an understanding of these motivations be integrated into psychosocial interventions to enhance and sustain caregiver wellbeing?

## Methods

### Research design and procedures

The current study draws qualitative dyadic interview data (*N* = 20) from a larger Randomized Controlled Trial for a novel Family Dignity Intervention (FDI) for Asian palliative care patients and their families (*N* = 50). The sampling methods, inclusion criteria, interview procedure and study protocol for the FDI are comprehensively described by Ho et al. [26]. Briefly, the FDI is developed based on an integrative body of empirical investigation that focuses on dignified end-of-life care in both Western and Asian contexts [27, 28]; it integrates elements of logotherapy and narrative life review to provide psycho-socio-spiritual support to patients and families facing mortality, and has been piloted for acceptability and feasibility before being fully adapted into the intervention study. In practice, FDI comprises a recorded dyadic semi-structured interview with a patient and a family caregiver conducted in their homes. The FDI therapist uses a guided question framework to facilitate joint conversation on shared memories and living wisdoms that lead to meaning-making and the expression of appreciation and reconciliation. This is done with the ultimate goal of creating a *legacy document* that tells the life story of the patient and is bestowed to the rest of the family through an open reading. Each interview lasted between 60 and 90 min and was conducted in English, Malay, Mandarin or a Chinese dialect (Hokkien, Teochew or Cantonese). These recorded interviews were transcribed verbatim, translated into English by a native language speaker where applicable, and edited into legacy documents. Transcripts and legacy documents were reviewed and finalized by patients and caregivers to ensure accuracy and authenticity.

### Sampling

The sample drawn for this study consisted of 20 primary family caregivers of older palliative care patients (aged 50 and above) with mainly a cancer prognosis of less than 12 months. Eleven were spousal caregivers, seven were adult-children caregivers, and two were sibling caregivers; the majority were female aged between 23 to 82, with a mean age of 56.2 years (see Table 1 for caregiver demographics). They were recruited through the in-patient, day-care, and homecare hospice service units of HCA Hospice Care, Dover Park Hospice, Tan Tock Seng Hospital, Singapore Cancer Society and Methodist Welfare Services. The inclusion criteria required family caregivers to be above 21 years old and identified by the patient to be their primary carer. Patients and family caregivers came from various socioeconomic backgrounds, and were predominantly of Chinese ethnicity. As the FDI focused on patient narratives, transcripts bearing a moderate to sizeable amount of input from the family caregiver were selected for data analysis.

**Table 1:** Demographics of Family Caregivers

Identifier	Caregiver Relationship	Caregiver Ethnicity	Patient’s Diagnosis	Patient’s Prognosis (Months)
DPH14	Child	Chinese	Lung CA	7–12
DPH19	Spouse	Chinese	Prostate CA	6
DPH34	Spouse	Chinese	Lung CA	2–3
DPH42	Sibling	Chinese	Sigmoid CA	2–3
DPH53	Spouse	Chinese	Lung CA	2–3
DPH59	Child	Chinese	Gynaecological malignancy	12
DPH68	Spouse	Malay	Lung CA	4–6
HCA12	Spouse	Eurasian	Prostate CA	2–3
HCA68	Child	Chinese	Colon CA	4–6
HCA75	Child	Malay	Breast CA	4–6
HCA81	Child	Chinese	Endometrial CA	12
HCA87	Spouse	Malay	Renal CA	12
HCA109	Spouse	Chinese	Endometrial CA	6
HCA114	Spouse	Chinese	Brain CA	4–6
HCA116	Spouse	Chinese	Nasopharyngeal CA	6
HCA117	Spouse	Chinese	Pancreas CA	12
MWS004	Sibling	Chinese	COPD	12
SCS18	Spouse	Chinese	Liver CA	12
TTSH61	Child	Malay	Lung CA	12
TTSH65	Child	Chinese	Gynaecological CA	12

### Data analysis

This study adopted an interpretative phenomenological analysis (IPA) to investigate the internalized motivations of family caregivers in tending to a dying family member. The IPA is an approach in qualitative research that aims to provide insights into how an individual makes meaning out of a phenomenon [29]. It is important to note that as the current study draws qualitative data from the larger FDI study, no explicit questions about internalized caregiver motivations were asked. Reflections on motivations towards caregiving occurred organically throughout the interview transcripts and were identified by the researcher using IPA through a process of data reduction and data reconstruction.

First, Authors 1 and 2 screened all transcripts and selected those that had adequate input from family caregivers to be used for analysis. Authors 1 and 2 then conducted line-by-line coding to develop descriptive themes and analytical categories, conceptualizing new interpretation of the data. This was followed by regular meetings among all authors for the further refinement of themes and categories to encapsulate the meaning and content within the cluster of similar codes, with the emergent themes and sub-themes created via a summary chart. All authors reviewed and defined the emergent themes; once consensus was reached, operational definitions were created. Finally, relationships between categories, themes and sub-themes were proposed and mapped with supporting quotes from transcripts. To address issues of trustworthiness and credibility, emergent themes were constantly compared and contrasted within and across groups during regular meetings, the final theme categorization and definitions were agreed upon by the entire research team, and data saturation and investigator triangulation were achieved [30].

## Findings

**Fig. 1:**
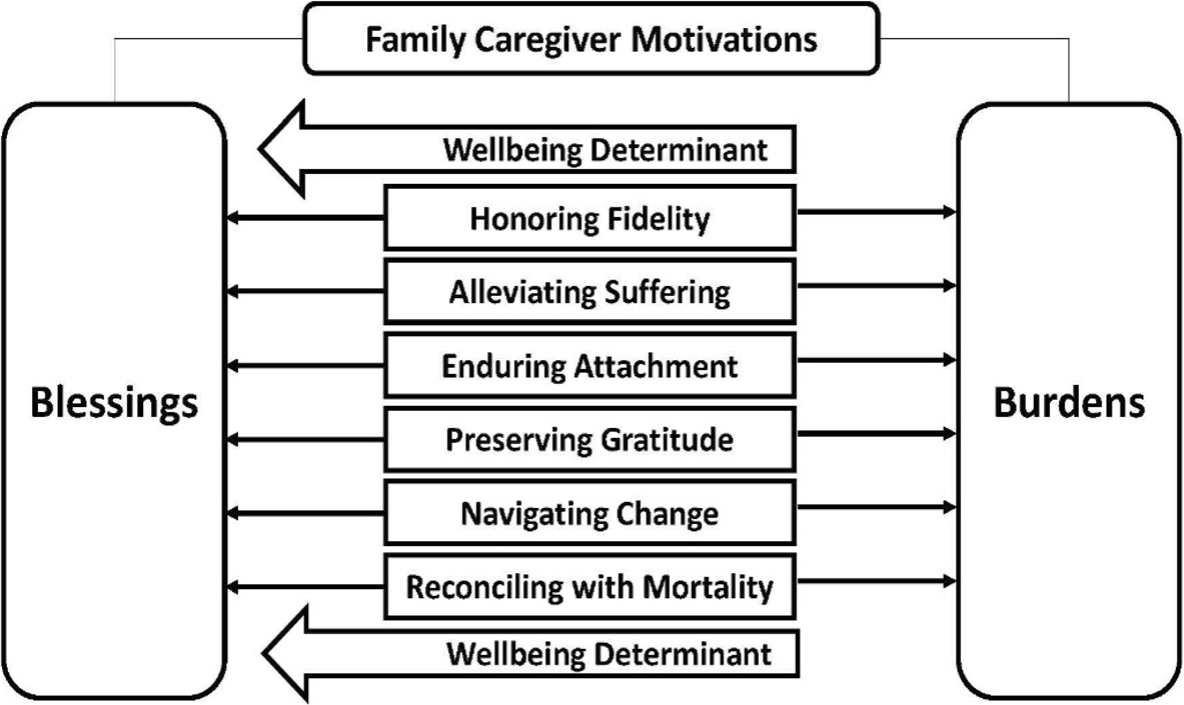
The Blessings or Burdens of End-of-life Caregiving (BoBEC) Model

Figure 1 presents the six caregiving motivations and one wellbeing determinant generated from data, to form the Blessings or Burdens of End-of-life Caregiving (BoBEC) model.

The six caregiving motivations (*Honoring Fidelity, Alleviating Suffering, Enduring Attachment, Preserving Gratitude, Navigating Change* and *Reconciling with Mortality*) represent the beliefs, values and goals that are assimilated into the EoL family caregiver’s daily life; each motivation is posited to affect the way a caregiver makes meaning of their role, hence leading to a nurturance of caregiver wellbeing (termed in this paper as *blessings*) or a diminishment in caregiver wellbeing (termed in this paper as *burdens*). The *Wellbeing Determinant*, which is characterized by the caregiver’s sense of control, self-empowerment and kinship derived from their experiences, serves as an indication of self-determination, and is theorized to have a positive influence on how the six caregiving motivations are appraised by the caregiver.

These themes are described in greater detail and demonstrated by direct quotes from study participants below.

### Honoring Fidelity (number of transcripts theme has occurred in; *N* = 13)

Family caregivers expressed their faithfulness and commitment to attend to the needs and wishes of their family members for the remainder of their lives, fearing regret to see things through till the end. This motivated them in fulfilling their sense of duty to the utmost of their abilities.

*“I shouldn’t regret anything. Whatever I can do for him, I will do my best and, [instead of waiting till] he’s in the coffin, you know, [and then say], ‘Oh, why didn’t I do this, why didn’t I do that?’ ” (DPH19, Spouse)*

In some instances, family caregivers displayed poignant emotion and dedication to their family members, conveying the great extent to which they would go to give their family members the best care and comfort.

*“I wish to care for him till the very end… I want the best for him and I will do what’s best for him. I am willing to sacrifice my soul to make that happen, or take his place if I could.” (DPH68, Child)*

### Alleviating Suffering (*N* = 13)

Family caregivers displayed awareness and empathy towards their family member’s physical and emotional suffering, tied in with a desire to relieve their pain.

*“This morning she was upset with me for forcing her to drink the bitter medicine. I told her, ‘I love you. I wouldn't do this if I had a choice. I want you to drink this for your own benefit, not mine. I'm just encouraging you from the side-lines.’ ” (DPH53, Spouse)*

Underlining this motivation to alleviate suffering was the family caregivers’ innate compassion for their family member, an empathic bond so strong that witnessing their family member’s suffering caused them deep emotional distress.

*“… It hurts a lot to drain the fluids. I’m heartbroken when I see how much pain she is in, especially when I see the tubing being inserted. It must hurt so much.” (HCA109, Spouse)*

### Enduring Attachment (*N* = 16)

Family caregivers experienced a prevailing attachment to their family member that motivated them in spending cherished time together and doing all they could to ensure that their family member was well taken care of.

*“I think I try to make him as comfortable as he can be. Every medical check-up, every appointment, we will keep to it, and I will always be there for him. [There will never be] any appointment that I am not going with him.” (HCA117, Spouse)*

Some family caregivers found that the motivation to sustain this attachment was also driven by feelings of anxiety about their family members’ wellbeing. These caregivers felt the need to be within their family members’ physical proximity as much as possible.

*“I get worried when she’s lying there and sleeping, because I’m not sure if anything has happened to her. I’m much happier when she’s sitting here with me. When she’s just lying there, I would think, ‘Oh no, what if something has happened to her?’ and I’d be worried.” (DPH59, Child)*

### Preserving Gratitude (*N* = 19)

Family caregivers described past circumstances and beliefs that motivated present feelings of gratitude to their family members. This consequently influenced their efforts and responses in caregiving.

*“She was constipated for as long as a week, and she didn’t tell me. When she eventually relieved herself, she made a mess on the bathroom floor. As I was cleaning the mess, I thought about how she had cleaned me up when I was little, so I didn’t mind.” (HCA81, Child)*

While many family caregivers reported feelings of gratitude stemming from how their family member had treated them in the past, some indicated that religious and cultural beliefs had indoctrinated a sense of indebtedness to their family members.

*“My mother says I was indebted to my brother in my past life; this is why I have to settle my debt in this lifetime [by caregiving], because he is here to get his compensation.” (MWS004, Sibling)*

### Navigating Change (*N* = 16)

Family caregivers reflected on their perceptions of the changes that had taken place in their lives and that of their family members throughout the illness trajectory. Some caregivers found motivation in helping their family members adapt to changes by dedicating time and energy to lift their spirits and provide emotional support.

*“I would bring my father food when I visit, while my husband would share words of encouragement and talk to him to cheer him up. We just want him to be happy, so that he wouldn't spend the whole day in negativity.” (TTSH65, Child)*

Others saw the changes as a temporary setback and found motivation in steering their family member back to their previous condition, if possible.

*“Sometimes I will move his legs a little, to give him that exercise. I hope that he can walk again, but it depends on how strong his will is.” (HCA116, Spouse)*

### Reconciling with Mortality (*N* = 18)

The knowledge of their family member’s prognosis motivated some family caregivers to make the most of the time left with their family members – creating treasured memories and remembering their legacies.

*“All of us just want to cherish the time that we have left with her, and we want her to help us spend more good times together. We [want to] learn about my grandmother, learn about my mother, so that we can pass on to the next generation; share with them the traits and the role models to look up to.” (TTSH61, Child)*

Other family caregivers perceived their family member’s prognosis to be unacceptable, choosing to push for further treatment in order to stall death’s journey to their doors.

*“My grandmother lived past 80 years old, so I thought my mother would live till at least 90 without any problems. I felt really shocked. Because I always thought, “She still has more than 10 years; I still have time.” … So we felt that, if it was possible, she should extend her life.” (DPH14, Child)*

### The Wellbeing Determinant (*N* = 11)

Family caregivers reflected on the discovery of positive changes amidst the trials of their family member’s illness. One such change was found within strengthened kinship.

*“As we grow up, it’s a bit harder [to have family gatherings] because we are all working. So when the disease came, even though it’s not a good thing, not something you will ask for, it united us again. Maybe without it, [we] would have been a bit more separated.” (TTSH61, Child)*

*“I feel like we are more united now. Maybe in the past we didn't really chat with each other… The amount of communication we had increased. I feel that our unity has become stronger.” (DPH14, Child)*

They expressed pride in overcoming initial fears by taking charge of and learning to perform unfamiliar tasks, with a newfound confidence in their abilities to face both practical and emotional difficulties. Family caregivers also demonstrated a sense of self-empowerment and strength-based reflection in their sharing.

*“I know nothing about going to visit the government… Or to do this, do that. But somehow, I find my way there. [I am a] much stronger person. So if anything happens to me, I think, I know, I can face up to it.” (DPH19, Spouse)*

*“This is how you grow. I learnt to grow because of [my husband]. You have to face the insurmountable challenges that come your way. I learnt how to shoulder my responsibilities on my own.” (SCS18, Spouse)*

## Discussion

This is the first known study that investigates and brings attention to the internalized motivations of EoL family caregivers. While the Family Dignity Intervention study questions did not specifically query family caregivers on their motives, these motivation-centred responses occurred spontaneously and abundantly throughout the interviews – an indication that internalized motivations are profoundly espoused within EoL caregiving attitudes and behaviors. The BoBEC Model (Fig. 1) illustrates the duality of these caregiving motivations in regard to meaning-focused coping [20] and intrapsychic strains [21], as well as identifies an important influence on caregiver wellbeing.

### Motivations with cultural influences

Some themes revealed cultural undertones that reflected the internalization of Asian values into family caregiver motivations. In their motivation for *Alleviating Suffering*, family caregivers displayed the desire to do so by practical means, such as administering medication to their family member, and experiencing distress when such methods were not feasible. This is in line with the Asian culture of preferring to show concern for their family members through pragmatic ways [26]. In their motivation for Preserving Gratitude, family caregivers demonstrated the significance of filial piety, as well as cultural beliefs about karma and past life [31], within their attitudes towards caregiving. Finally, in their motivation for Reconciling with Mortality, family caregivers indicated the importance placed on close intergenerational connections as well as longevity for their elders [28].

### Motivations as blessings: Meaning-focused coping

All EoL caregiving motivations (*Honoring Fidelity, Alleviating Suffering, Enduring Attachment, Preserving Gratitude, Navigating Change* and *Reconciling with Mortality*) were found to embody the tenets of meaning-focused coping. Fuelled by these motivations, family caregivers displayed the propensity for benefit-finding and benefit-reminding even in witnessing their family member’s suffering and imminent mortality; adaptive goal processes in adjusting their expectations and aspirations in accordance with their family member’s physical condition and prognosis; reordering priorities in hopes of making the most of the time left with their family member; and infusing ordinary events, both past and present, with positive meaning that allowed them to feel affirmed, encouraged and grateful in their daily caregiving [20]. As such, the capacity for imbuing stressful caregiving events with positive meaning and responses would make these motivations advocates of perceived “blessings” in the EoL caregiving journey.

### Motivations as burdens: Intrapsychic strains

Paradoxically, the authors found that these EoL caregiving motivations also ran parallel to the intrapsychic strains as postulated by Pearlin and colleagues [32] in their seminal Stress Process Model. Intrapsychic strains occur when the caregiver’s self-concept is diminished due to the chronicity of providing care. Intrapsychic strains unique to caregivers were defined as: 1) *role captivity*, in which the caregiver feels entrapped within his or her role whether or not by personal choice, 2) *the loss of self*, in which the caregiver experiences a loss of identity and sense of personhood as enmeshment with the patient ensues, 3) *perceived low competence*, in which the caregiver does not see the value and skill of the work they do, leading to a sense of helplessness, and 4) *perceived lack of gain*, in which the caregiver does not find personal growth or enrichment in the caregiving process.

Should their EoL caregiving motivations personify these strains, it is only a matter of time before family caregivers experience outcomes of mental and emotional distress, such as depression, anxiety, and irritability, as well as a decline in physical health and a disengagement from their caregiving roles [32]. In short, these motivations would most certainly bludgeon the family caregiver with great ‘burdens’ within the EoL caregiving journey.

### The crucial factor: Self-determination

Self-determination theory [33] suggests that people need to feel a sense of *competence* (gaining mastery of tasks and having self-efficacy), *relatedness* (feeling like they belong and mutually relating to others) and *autonomy* (control over their own choices, behaviors and goals) in order to fuel high quality motivation that helps one to thrive. The theme of the *Wellbeing Determinant* aligns with this concept in a caregiving-centric phenomenon. Family caregivers contributing to this theme displayed confidence in carrying out previously unfamiliar caregiving tasks (*competence*), affirmed a stronger sense of kinship (*relatedness*) with the patient and their families, and took ownership of their caregiving responsibilities and challenges (*autonomy*). Encouragingly, numerous studies have proposed that high quality motivations stemming from self-determination can elicit outcomes of greater fortitude, higher commitment, and more positive emotions and self-concepts [34–36].

Building on said studies, the authors propose that family caregivers who feel a sense of competence, relatedness and autonomy within their caregiving motivations would be further inclined to meaning-focused coping, such as 1) deriving perceived benefits from their caregiving even in difficult events, 2) reminding themselves of these benefits when faced with similar circumstances, 3) setting their own goals in caregiving, 4) having the competence and confidence to be flexible and adaptive and 5) finding positivity in normal, everyday situations. Possessing a sense of self-determination in the caregiving role would in essence safeguard one’s caregiving motivations from the intrapsychic strains of perceived entrapment, a sense of disempowerment, ineptitude and fruitlessness.

As such, the BoBEC model identifies the *Wellbeing Determinant* as an indicative element of caregiver self-determination and a crucial factor as to whether the EoL family caregiver perceives their journey as one lined with blessings or laden with burdens.

### Implications and recommendations

Findings from a number of studies show that fulfilling one’s sense of self-determination appears central to sustaining one’s motivation and innate satisfaction in caregiving [34–36]. The findings from this paper indicate that caregivers are driven by motivations that could equally contribute to wellbeing nurturance or diminishment. At the same time, our findings also indicate that caregivers possess a caregiver-centric sense of self-determination (the Wellbeing Determinant) that is theorized to have positive affect on their motivations. Thus, this paper recommends that caregiver support interventions should comprise all of the following mediators in order to fulfil the need for self-determination:

1. **Competence-targeted mediators**: Apart from general psychoeducation on symptom management, medical care and self-care, interventions could incorporate mediums to develop and improve self-efficacy. These can take the form of personal strengths journaling, facilitating peer support between new and experienced caregivers, role-modelling and goal-setting. Such interventions can be created in the form of structured support groups, online platforms or mobile applications.
2. **Autonomy-targeted mediators**: In addition to providing adequate and appropriate EoL caregiver education, autonomy-targeted interventions such as those involving mindfulness practice and the arts can serve to give caregivers a sense of control as well as help them make meaning out of their thoughts, emotions and circumstances. To date, the Mindful-Compassion Art Therapy (MCAT) for EoL care professionals [37] shows great potential to be converted into a program for EoL family caregivers.
3. **Relatedness-targeted mediators**: Dyadic or family projects that recall shared memories, express appreciation, seek forgiveness, impart wisdom and create generativity (such as the FDI) are valuable in crafting the bond of relatedness among family caregivers and their family members. Such projects should be implemented as a foundation of psychosocial interventions at the end-of-life.

Interventions should be offered to family caregivers in a culturally-relatable manner. This can be done through emphasizing how these mediators will help family caregivers enhance their practical caregiving with increased competence, sense of control and meaning-making, as well as enrich their intergenerational familial bonds with conversations that focus on legacy creation.

### Limitations and future directions

While the organically occurring responses emphasize the significance of motivations within EoL caregiving, these responses were not examined further during the FDI interviews due to other primary objectives. Future research specifically exploring intrapsychic EoL caregiving motivations would surely provide deeper insight into the matter, lending strength to more informed interventions. A more diverse sampling with stronger representation of populations would enhance moderatum generalization. Finally, the application of the BoBEC model and its clinical recommendations remain to be assessed for its precision and effectiveness in supporting EoL caregivers through future intervention studies.

## Conclusion

It is impossible to climb the ‘mountain’ of end-of-life caregiving [1] without fear nor misstep. No matter the vigour or stamina of one’s inherent motivations, the chronicity and strains of EoL caregiving may cause these motivations to diminish into a state of burden and moral entrapment. Yet, when one is hanging exhausted by their fingertips at a precipice, the ability to reclaim one’s sense of self-determination would nurture the strength and purpose needed to haul themselves up, dust themselves off and, in tired triumph, admire the blessings of caregiving amidst a beautiful view.

## Data Availability

All data collected during this study which can be made publicly available is included in this article. Additional data from the current study is not publicly available due to concerns about participants’ confidentiality.

## Abbreviations

EoL: End of Life
FDI: Family Dignity Intervention
